# Capillaroscopy as a diagnostic tool in the diagnosis of mixed connective tissue disease (MCTD): a case report

**DOI:** 10.1186/s41927-021-00179-2

**Published:** 2021-03-19

**Authors:** Mislav Radić, Rebecca S. Overbury

**Affiliations:** 1 Department of Rheumatology and Clınıcal Immunollogy, University of Split, Split, Croatia; 2 Department of Internal Medicine, Division of Rheumatology, University of Utah, 30 N 1900 E, Ste 4B200, UT 84132 Salt Lake City, USA

**Keywords:** Juvenile mixed connective tissue disease, Mixed connective tissue disease, Capillaroscopy, Raynaud’s phenomenon, Classification criteria

## Abstract

**Background:**

The concept of mixed connective tissue disease (MCTD) as a unique connective tissue disease has endured for half a century. Disease onset can be in adulthood (MCTD) or of juvenile onset (jMCTD) and is characterized by overlapping features of systemic lupus erythematosus (SLE), polymyositis or dermatomyositis (PM/DM) and systemic sclerosis (SSc). No universally accepted classification criteria for MCTD exists, however agreed upon overlapping disease features include the presence of high titers of U1 small nuclear ribonucleoprotein particle antibodies (U1snRNP) in the peripheral blood, Raynaud’s phenomenon, synovitis, myositis and swollen hands or fingers. Characteristic capillaroscopy findings are commonly seen in MCTD and jMCTD, which may represent a crucial and key clue for classification as well as prognosis in these patients.

**Case presentation:**

We present a young male patient, with symptom onset as early as age 13, who was diagnosed with MCTD at age 16 and found to have high titers of anti-U1snRNP antibodies, Raynaud’s phenomenon, synovitis, and swollen hands and fingers. Most interestingly, his video capillaroscopy at diagnosis was abnormal and revealed an active SSc-like pattern. His presentation and course are described.

**Conclusions:**

We conclude that based on existing data, and as highlighted by this case presentation, nailfold video capillaroscopy should be included as an early screening tool for the detection of microangiopathy in patients with the diagnosis MCTD and jMCTD. Additionally, given its prevalence in this population at disease diagnosis, we recommend consideration be given to nailfold video capillaroscopy as a potentially important classification criteria and prognostic tool for jMCTD and MCTD.

## Background

**Fig. 1:**
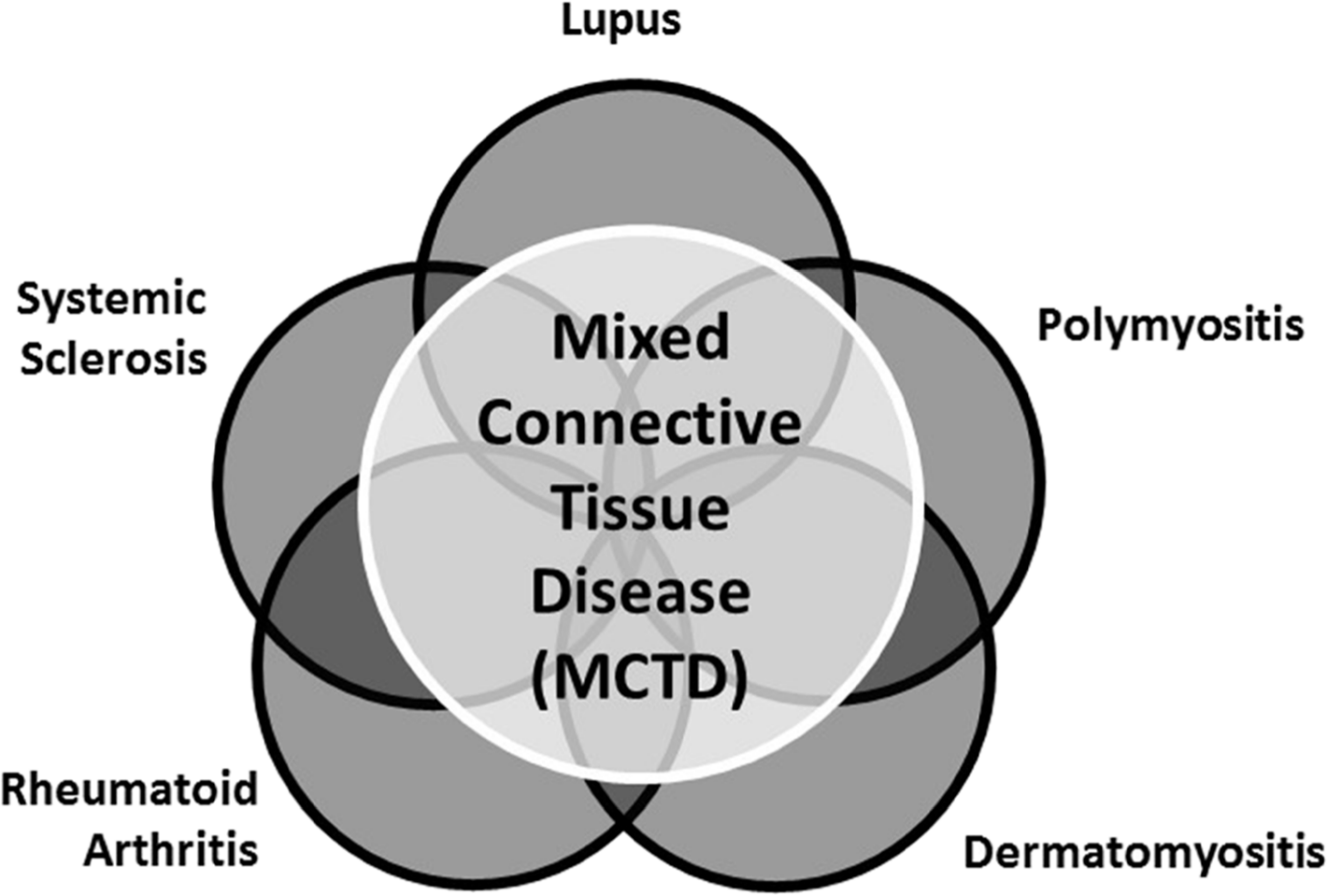
Mixed connective tissue disease concept

The concept of mixed connective tissue disease (MCTD) as a unique connective tissue disease has persisted for almost 50 years. MCTD was first described by Sharp et al. in 1972 [1]. Shortly thereafter, the initial reports of juvenile onset MCTD (jMCTD) emerged [2, 3]. MCTD and jMCTD are characterized by overlapping features of systemic lupus erythematosus (SLE), polymyositis (PM) and dermatomyositis (DM), and systemic sclerosis (SSc) with high titers of antibodies targeting the U1 small nuclear ribonucleoprotein particle (U1snRNP) in the peripheral blood. Subsequently, it was found that MCTD patients can also have rheumatoid arthritis (RA) manifestations with a similar inflammatory arthritis pattern (Fig. 1) [4]. A juvenile presentation, defined as disease onset prior to the age of 16, occurs in 7–23% of all MCTD cases [5, 6]. The female: male ratio in jMCTD is 6:1 [7].

The etiology of MCTD and jMCTD, like other systemic autoimmune rheumatic diseases, is unclear. Current models support a hypothesis involving chronic immune activation after exposure to an environmental or exogenous trigger in individuals with a predisposing genetic background. Certain major histocompatibility complex (HLA) genes have an important role in the presentation of antigens to the immune system. Specifically, HLA-DR4, HLA-DRw53, HLA-DRB1*04:01 and HLA-B*08 have all been found to play a role in the creation of anti-U1snRNP antibodies and ultimately the clinical manifestations of the disease [8–10].

The classification of MCTD that seems dependent on this single serological finding has fostered doubts that MCTD is in fact a unique disease entity [11, 12]. Some researchers theorize that MCTD represents instead an overlap syndrome or an early and unspecific phase of another defined connective tissue disease. However, the concept of MCTD as a defined entity is supported by the existence of a specific and repeated clinical pattern, a characteristic antibody, and associated specific immunological and genetic findings [13]. This pattern and the risk for similar end-organ disease manifestations appears similar in jMCTD and MCTD [14, 15].

MCTD definition further suffers from a lack of consensus regarding disease classification criteria. Multiple sets of classification criteria for MCTD have been published, including: Sharp [16], Kasukawa et al. [17], Alercón-Segovia and Villareal [18] and Kahn and Appeboom [19]. Regardless of their variability, the criterion common to all is the detection of anti-U1 RNP antibodies. In jMCTD, Kasukawa criteria are used most frequently in published series (Table 1) [20]. It is important to emphasize that all of these models constructed classification criteria for MCTD and not jMCTD.

**Table 1:** Classification criteria for mixed connective tissue disease

A. Common symptoms	
1. Raynaud’s phenomenon	
2. “Swollen”/“puffy” fingers or hands	
B. Anti-U1snRNP antibodies	
C. Miscellaneous	
**1. Lupus Manifestations**	
a. Polyarthritis	
b. Adenopathies	
c. Malar erythema	
d. Serositis (pleuritis or pericarditis)	
e. Cytopenia (leukopenia or thrombocytopenia)	
**2. Sclerodermiform manifestations**	
a. Sclerodactyly	
b. Pulmonary fibrosis, restriction or decrease of diffusion < 70% c. Hypomotility or oesophageal dilation	
**3. Myositis**	
a. Muscular weakness	
b. Muscle enzymes (CK) increase	
c. Myopathic pattern on EMG	

*CK* creatine kinase, *EMG* electromyography, *snRNP* small nuclear ribonucleoprotein

At least 1 of the 2 common symptoms is required, plus positive anti-U1snRNP antibodies, plus one or more of the miscellaneous symptoms in at least 2 of the 3 categories. Kasukawa et al. [17]

Perhaps the best attempt to describe jMCTD is as an undifferentiated connective tissue disease represented mostly by Raynaud’s phenomenon and anti-U1snRNP antibodies. The most commonly described symptoms of jMCTD are Raynaud’s phenomenon, polyarthritis or polyarthralgia, myositis, sclerodactyly, edema of the hands and fingers, and esophageal dysmotility. The most common disease characteristics in jMCTD are summarized in Table 2 [7].

**Table 2:** Frequency of clinical findings in juvenile onset mixed connective tissue disease [17]

Disease characteristics	Frequency
Raynaud’s phenomenon	+ + + +
Arthritis	+ + + +
Muscle disease	+ + +
Fever	+ + +
Lung disease (often mild at onset)	+ + +
Thickened skin of scleroderma	+ + +
Dry eyes and dry mouth	+ +
Rash of lupus (SLE)	+ +
Rash of juvenile dermatomyositis	+ +
Central nervous system disease	+
Heart disease	+
Pulmonary hypertension	+
Kidney disease	+ / –

*SLE* systemic lupus erythematosus

There are no treatments available specific to MCTD. According to the first descriptions of the disease, MCTD patients were characterized by an excellent response to glucocorticoid treatment and a favorable prognosis [4]. However, therapy needs to be individualized and adapted according to the severity of the manifestations at the time of presentation and organ involvement.

As stated, Raynaud’s phenomenon, which can be primary (idiopathic) or secondary, is a major clinical feature of MCTD and jMCTD. Nailfold videocapillaroscopy is a non-invasive, inexpensive, and reproducible imaging method allowing the evaluation of structural changes in the peripheral microcirculation that can be associated with Raynaud’s phenomenon. The most important indications for performing capillaroscopy include determining primary versus secondary Raynaud’s phenomenon, as well as assessment and diagnosis of scleroderma spectrum disorders. Nailfold capillaroscopy findings are usually classified as normal, non-specific, or scleroderma-like (SSc-like) [21]. In MCTD, Raynaud’s phenomenon may precede the development of additional symptoms and so capillaroscopy provides an ideal opportunity to diagnose the earliest stages of damage to the microcirculation.

In this case report we present a patient diagnosed with MCTD in whom capillaroscopic findings at diagnosis revealed SSc-like capillaroscopic disease manifestations.

## Case presentation

At the age of 13 our Caucasian male patient, a competitive baseball player, living with his biological parents and siblings, suffered the progressive onset of right shoulder, arm, and hand pain. Although, there was no preceding trauma, since he was a baseball pitcher, he visited various medical providers with the understanding that this was secondary to a baseball related injury. He underwent 6 months of physical therapy, yet the shoulder pain only progressed. He was not referred to rheumatology. Three years later, with persistent and progressive symptoms, the patient presented again to the medical system with persistent pain, redness, and swelling of his right elbow and right hand. However, by now the symptoms had also progressed to include pain, swelling and stiffness, especially in the morning, of the bilateral fingers, hands, elbows, shoulders, hips, knees, and ankles.

He was referred to our adolescent rheumatology clinic in December 2018 for assessment of these symptoms. He was at the time of our first assessment,16-years-old, 66 kg, 182 cm (BMI 20 kg/m^2^). Following specific inquiry and review of systems, he also endorsed long-standing difficulty with gastrointestinal symptoms (including certain food intolerances, abdominal pain and cramping, diarrhea, rare nausea and occasional dysphagia) for which previous providers had diagnosed him with irritable bowel syndrome. In addition, the patient confirmed symptoms of Raynaud’s phenomenon that included triphasic color changes with associated pain of years duration. On examination, he had normal vital signs for his age and inflammatory arthritis. Specifically, he had a reduced and painful jaw excursion; pain on motion in the right shoulder; arthritis with reduced range of motion in the right elbow; bilateral swollen hands with arthritis of the bilateral wrists, multiple bilateral metacarpal-phalangeal joints, multiple bilateral proximal interphalangeal joints, bilateral knees, and bilateral ankles; and tenderness without obvious inflammation in the bilateral feet and metatarsal phalangeal joints. His skin exam, excluding capillaroscopy described below, was normal without any digital pits or ulcers and without rash, skin tightening, nodules, calcinosis, or mucosal ulceration. Muscle strength was normal. The remainder of his examination including cardiopulmonary and gastrointestinal systems was normal.

**Fig. 2:**
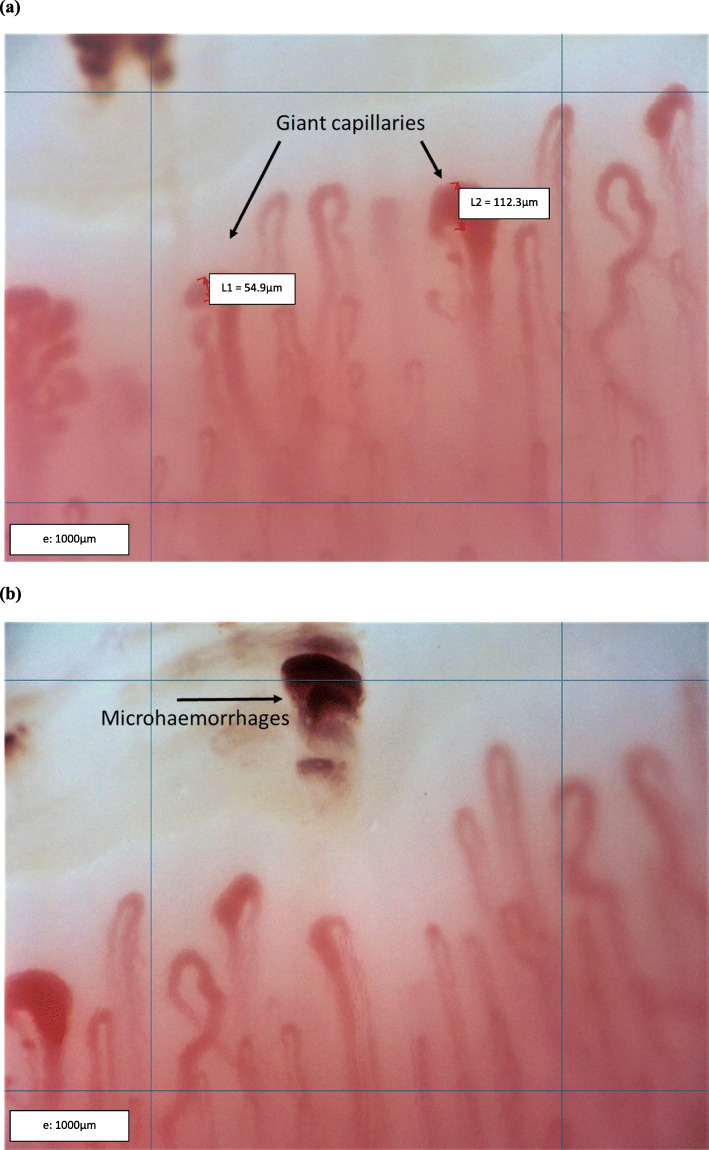
‘Active’ scleroderma pattern, giant capillaries (**a**), reduced capillary density and hemorrhages (**b**)

His nailfold video-capillaroscopy revealed an ‘active’ scleroderma pattern (Fig. 2). Nailfold video-capillaroscopy was performed in a quiet, temperature-controlled room (22–24 °C) according to previously published expert recommendations [22]. The nailfolds of the bilateral second, third, fourth, and fifth fingers were examined and analyzed by our investigator (MR) who was blinded to the results of biologic and radiologic assessments. Nailfold examination and analysis was performed using optical probe videocapillaroscopy equipped with a 200x magnification contact lens and connected to image analysis software (Inspectis AB, Solna, Sweden).

Serologic analysis included an elevated anti-nuclear antibody (ANA) and anti-U1-snRNP antibody with an otherwise negative result for other extractable nuclear antigen and other clinically relevant antibodies (Table 3). Complement was normal. Inflammatory markers were only mildly elevated. CBC with full differential and metabolic panel were normal. Urine analysis was bland without protein, red blood cells, white blood cells, or casts. Radiographs of the chest, bilateral feet, and bilateral hands were normal with normal bone density and preserved joint spaces. Radiographs of bilateral knees were with small to moderate effusions but otherwise normal. Infectious disease screening including HIV, hepatitis B, hepatitis C, and tuberculosis was negative. He was diagnosed with MCTD, with presumed juvenile onset given the duration of his undiagnosed symptoms [23].

**Table 3:** Serologic and laboratory data

Lab	Result	Normal Range
ANA, HEp-2, IgG	> 1:2560 (speckled)	< 1:40
Smith-RNP Ab, IgG	135 AU/mL	0–40 AU/mL
Smith Ab, IgG	5 AU/mL	0–40 AU/mL
dsDNA Ab, IgG	none detected	none detected
SSA (Ro) Ab, IgG	Negative	0–40 AU/mL
SSB (La) Ab, IgG	3 AU/mL	0–40 AU/mL
Scl-70 Ab, IgG	6 AU/mL	0–40 AU/mL
Centromere Ab, IgG	3 AU/mL	0–40 AU/mL
RNA polymerase III Ab, IgG	8 units	0–19 units
Beta-2-glycoprotein Ab, IgG/IgM	1 SGU/3 SMU	0–20 SGU/0–20 SMU
Cardiolipin Ab, IgG/IgM	3 GPL units/3 MPL	0–14 GPL units/0–12 MPL
CCP Ab, IgG	< 0.5 unit/mL	< 0.5 unit/mL
Rheumatoid factor	< 9 IU/mL	< 9 IU/mL
ESR	4 mm/h	0–15 mm/h
CRP	2.1 mg/dL	0–0.7 mg/dL
C3	112 mg/dL	83–152 mg/dL
C4	13 mg/dL	13–37 mg/dL
Creatine kinase	57 unit/L	55–370 unit/L

*ANA* antinuclear antibody, *HEp-2* human epidermoid cancer cells, *RNP* ribonucleoprotein, *Ab* antibody, *dsDNA* double-stranded deoxyribonucleic acid, *SSA* Sjögren’s syndrome related antigen A, *SSB* Sjögren’s syndrome related antigen B, *Scl-70* topoisomerase I, *RNA* ribonucleic acid, *CCP* cyclic citrullinated peptide, *ESR* erythrocyte sedimentation rate, *CRP* c-reactive protein, *C3* complement component 3, *C4* complement component 4

He was started on subcutaneous methotrexate with folic acid (increased over several weeks to 25 mg subcutaneously given weekly), hydroxychloroquine 300 mg daily (5 mg/kg/day) and oral glucocorticoids 20 mg daily. He had rapid symptomatic improvement. High-resolution computed tomography (CT) scan of the chest, complete transthoracic echocardiogram (TTE), and pulmonary function tests (PFT) over the next 8 weeks were normal. Ophthalmological examination was normal. Oral glucocorticoids were slowly tapered, at intervals of 5 mg every few weeks, and he was completely weaned by early April of 2019. As of September 2019, despite apparent medical compliance, he was developing refractory arthritis similar to his original presentation, mild muscle ache with a normal creatinine kinase, worsening Raynaud’s phenomenon, and unchanged capillaroscopy abnormalities. His therapy was escalated to include abatacept (125 mg subcutaneously given weekly) and extended release nifedipine (30 mg daily). These agents were chosen to target his ongoing active synovitis and Raynaud’s phenomenon. While tumor necrosis factor *a* inhibition (TNF*i*) was considered, given the possibility of systemic lupus erythematosus features in MCTD, we did not want to risk drug-induced lupus symptoms from a TNF*i* therapy. Pulmonary function tests and echocardiogram have not yet been repeated. To date he has developed no additional signs or symptoms. There have been no additional changes in routine laboratory monitoring.

## Discussion and conclusions

There is no international consensus on how, when, and in whom MCTD should be diagnosed as there is no current consensus regarding the disease classification criteria. Generally, the classification of rheumatic diseases is quite challenging because of protean and frequently overlapping clinical and laboratory manifestations [24, 25]. The purpose of classification criteria, of course, are to identify patients with a similar clinical entity for research, and classification criteria are not synonymous with diagnostic criteria. Additionally, although classification criteria will usually mirror those used clinically for diagnosis, classification criteria are generally more standardized, less inclusive, and thus less sensitive compared to clinical diagnosis [26, 27]. In the absence of a single diagnostic test that can define or confirm MCTD, four sets of classification criteria have been developed [16–19]. There has been no evidence-based or cultural consensus on a single accepted classification criterion for MCTD and so all should be considered [27]. Similarities between the criterion include that MCTD is likely in an anti-U1snRNP-positive patient presenting with Raynaud’s phenomenon and diffuse hand edema (“puffy hands”) with at least two of the following features: arthritis, myositis, leukopenia, esophageal dysmotility, pleuritis, pericarditis, interstitial lung disease or pulmonary hypertension. Raynaud’s phenomenon may precede the development of additional symptoms of MCTD and Raynaud’s phenomenon with “swollen” or” puffy” fingers or hands is a key clinical criteria in all recent classification criteria (Table 4) [16–19]. Recent study indicates that the classification criteria of Kasukawa et al. are the most sensitive (75%) compared to those of Alarcon-Segovia and Villarreal’s (73%) and Sharp’s (42%) in classification of patients with MCTD, throughout disease progression [28].

**Table 4:** Distribution and importance Raynaud’s phenomenon and “swollen”/“puffy” fingers or hands in the four published MCTD criteria sets [16–19]

	Kasukava et al.	Sharp	Alarcón-Segovia and Villareal	Kahn and Appelboom
Raynaud’s phenomenon	one of two obligatory criteria	one of four major criteria	one of five clinical criteria	obligatory criteria
“swollen”/“puffy” fingers or hands	one of two obligatory criteria	one of four major criteria	one of five clinical criteria	one of three clinical criteria

In a systemic disease in which vascular damage is one of the pathogenetic factors, abnormalities in capillary morphology should be observed long before the onset of clinical symptoms. In patients already diagnosed with a vasculopathic systemic disease, damage to the capillaries may reflect the involvement of internal organs and help determine the stage of the disease [29]. A long history of Raynaud’s phenomenon is usually reported in MCTD patients, even prior to diagnosis, and Raynaud’s phenomenon is often the only, or one of few, MCTD symptoms at the time of suspected diagnosis [5].

Notably, capillaroscopy has been included in the updated 2013 American College of Rheumatology/ European League Against Rheumatism (ACR/EULAR) classification criteria for SSc (a disease with phenotypic overlap to MCTD and a similarly understood vascular pathology and microangiopathy) and is considered a key investigative tool in the early phase of the disease [30, 31]. In 95% of SSc patients, peripheral microangiopathy follows a typical scleroderma pattern, consisting of ‘early’ (combination of few enlarged/giant capillaries, few capillary microhemorrhages, a relatively well-preserved capillary distribution, and no evident loss of capillaries); ‘active’ (frequent giant capillaries, frequent capillary microhemorrhages, moderate loss of capillaries, mild disorganization of the capillary architecture, and absent or mild ramified capillaries);and ‘late’ (few or absent giant capillaries and microhemorrhages, severe loss of capillaries with large avascular areas, and presence of neoangiogenesis defined by irregular enlargement of the capillaries, disorganization of the normal capillary array, and ramified/bushy capillaries) phases [32].

MCTD similarly seems to have a pattern of microangiopathy. Granier et al. reported a scleroderma-like pattern in 64% of MCTD patients, with bushy capillaries as a dominant feature [33]. Giant capillaries are another dominant feature observed in 54% of MCTD patients [34]. The sensitivity of giant capillaries’ presence, one of the most characteristic capillaroscopic findings, has been estimated to be 56% for MCTD [35]. In a recent study, a plurality (44%) of MCTD patients have demonstrated an ‘early’ scleroderma pattern on capillaroscopy [36]. Another study found that the ‘early’ pattern was associated with a positive anti-RNP antibody [37]. Interestingly, MCTD might have a differing underlying microvascular pathophysiology with less avascular areas or capillary drop-out compared to patients with SSc [37]. In a large prospective study with 3029 patients with primary Raynaud’s phenomenon (mean follow up period of 4.8 years) a scleroderma-like pattern was also significantly associated with the development of MCTD, and the number of MCTD patients with the scleroderma pattern on capillaroscopy increased over time [38, 39]. Additionally, recent study has shown that giant capillaries, as seen in our patient, might be a promising marker for interstitial lung disease in MCTD patients, especially among those with a short disease duration [40]. This is particularly interesting as it could suggest peculiarities of MCTD related pulmonary disease that point to an independent disease pattern.

Similar to MCTD, jMCTD is usually described as beginning with polyarthritis, Raynaud’s phenomenon, hand edema (“puffy hands”), and sclerodactyly. Later in the disease course, jMCTD clinical manifestations can include esophageal dysmotility, nervous system manifestations, pulmonary hypertension, and interstitial lung disease [7]. Typical findings of systemic lupus erythematosus (SLE) and PM or DM-like features are more evident at the time of diagnosis in jMCTD compared to MCTD, and several studies have confirmed that SLE-like features are more common among jMCTD patients compared to adult onset disease [6, 7, 41, 42]. The frequency of these manifestations however decreases during the course of the disease, giving way to the prevalence of SSc-like manifestations. Furthermore, jMCTD patients with SSc-like features seem to have a higher mortality in the case of internal organ involvement, and SSc-like changes may appear as long as 10 years after the initial diagnosis of jMCTD. Hetlevik et al. reported that all patients with jMCTD had Raynaud’s phenomenon early in the disease course, and that 94% had this clinical manifestation during the mean follow-up period of 16.2 years [41]. In this study the most common disease manifestations were Raynaud’s phenomenon (100%), arthritis (94%), puffy hands (77%) and pulmonary manifestations (58%). In a French cohort of 19 jMCTD patients, with a mean follow-up of 3.2 years, the most common disease manifestations were: arthritis (100%), Raynaud’s phenomenon (84%), pulmonary manifestations (47%) and sclerodactyly (42%). In more than half of these jMCTD patients with Raynaud’s phenomenon pathological nailfold capillaroscopic findings have been demonstrated (enlarged capillary loops, loss of capillary loops with avascular areas and neoformation of capillaries) [43]. Overall, more than half of patients with MCTD and jMCTD demonstrate a ‘scleroderma pattern’ on nailfold capillaroscopy, and this has been reported to be associated with the development of internal organ complications. Specifically, scleroderma-like abnormalities in MCTD often (76% of patients) accompany interstitial lung disease (ILD) [44].

Consistent with this described presentation, our patient presented to our adolescent rheumatology clinic with previously unrecognized Raynaud’s phenomenon and subsequent nailfold video-capillaroscopy which confirmed an ‘active’ SSc pattern. This has guided and informed his work-up and will continue to do so moving forward. Nailfold capillary changes in MCTD and jMCTD seem to be an early identifiable and dynamic process that could predict end-organ disease manifestations. Hence, we argue that nailfold video-capillaroscopy, which is the gold standard for detection of microvascular abnormalities and already a critical component of the systemic sclerosis classification criteria, should be considered as an early screening tool for the detection of microangiopathy in patients with the diagnosis of MCTD and jMCTD. Additionally, given its prevalence in this population at disease diagnosis, we recommend consideration be given to nailfold video-capillarscopy as a potential classification criterion for jMCTD and MCTD in the future.

In summary, based upon the existing research in this area, and as outlined in our above case, we propose that nailfold video capillaroscopy be included in the early assessment of all patients with MCTD and jMCTD. It should be considered in future classification criteria, as a screen for early detection of disease, and as part of routine follow-up for patients diagnosed with or who present with suspicion for MCTD and jMCTD.

## Data Availability

The dataset used in the current study are available from the corresponding author on reasonable request.

## Abbreviations

MCTD: Mixed connective tissue disease
jMCTD: Juvenile mixed connective tissue disease
SLE: Systemic lupus erythematosus
PM: Polymyositis
DM: Dermatomyositis
SSc: Systemic sclerosis
U1snRNP: U1 small nuclear ribonucleoprotein particle
RA: Rheumatoid arthritis
HLA: Human leukocyte antigen
Kg: Kilogram
Cm: Centimeter
BMI: Body mass index
°C: Degrees Celsius
ANA: Anti-nuclear antibody
CBC: Complete blood count
HIV: Human immunodeficiency virus
Mg: Milligrams
CT: Computed tomography
TTE: Transthoracic echocardiogram
PFT: Pulmonary function tests
TNF*i*: Tumor necrosis factor *a* inhibitor
ACR: American College of Rheumatology
EULAR: European League Against Rheumatism
ILD: Interstitial lung disease
